# Enhancing Preparedness for Arbovirus Infections with a One Health Approach: The Development and Implementation of Multisectoral Risk Assessment Exercises

**DOI:** 10.1155/2020/4832360

**Published:** 2020-04-20

**Authors:** Maria Grazia Dente, Flavia Riccardo, Wim Van Bortel, Laurence Marrama, Thomas Mollet, Tarik Derrough, Bertrand Sudre, Paolo Calistri, Gloria Nacca, Alessia Ranghiasci, Camille Escadafal, Lobna Gaayeb, Ariane Guillot, Miguel Angel Jiménez-Clavero, Jean-Claude Manuguerra, Guillain Mikaty, Marie Picard, Jovita Fernández-Pinero, Elisa Pérez-Ramírez, Vincent Robert, Kathleen Victoir, Silvia Declich

**Affiliations:** ^1^Istituto Superiore di Sanità, Rome, Italy; ^2^European Centre for Disease Prevention and Control (ECDC), Stockholm, Sweden; ^3^Institute of Tropical Medicine, Antwerp, Belgium; ^4^Research Executive Agency, European Commission Brussels, Brussels, Belgium; ^5^Istituto Zooprofilattico Sperimentale dell'Abruzzo e del Molise (IZSAM), Teramo, Italy; ^6^Institut Pasteur, Paris, France; ^7^FIND (Foundation for Innovative New Diagnostics), Geneva 1202, Switzerland; ^8^Centro de Investigación en Sanidad Animal (INIA-CISA), Valdeolmos, Madrid, Spain; ^9^CIBER Epidemiología y Salud Pública (CIBERESP), Madrid, Spain; ^10^MIVEGEC Unit, University of Montpellier, IRD, CNRS, Montpellier, France

## Abstract

**Background:**

One Health is receiving attention for arbovirus infection prevention and control and for defining national “intersectoral” priorities. Increasing awareness of intersectoral priorities through multisectorial risk assessments (MRA) is promising, where data are not systematically shared between sectors. Towards this aim, the MediLabSecure project organized three MRA exercises (hereby called exercises): one on West Nile virus, one on Crimean–Congo haemorrhagic fever, and one on Rift Valley fever, assessing the added value of this approach.

**Methods:**

The exercises relied on RA methodologies of international organisations. Country representatives of the human and animal virology, medical entomology, and public health sectors (hereby called “sectors”) involved in the surveillance of vector-borne diseases participated in the exercises. Background documentation was provided before each exercise, and a guide was developed for the facilitators. All three exercises included technical and methodological presentations and a guided RA directed at bringing into play the different sectors involved. To assess the added value of the approach, each participant was asked to rank the level of perceived benefit of the multisectoral collaboration for each “risk question” included in the exercises.

**Results:**

In total, 195 participants from 19 non-EU countries in the Mediterranean and Black Sea regions took part in the exercises. The participants assessed the multisectoral approach as valuable in analysing comprehensively the situation by having access to information and knowledge provided by each of the sectors involved. Sharing of information and discussion facilitated reaching a consensus on the level of risk in each country.

**Conclusions:**

Increasing awareness of intersectoral priorities, including cross-border ones, through MRA is relevant to reduce gaps due to unavailability of shared data and information. Given that six out of the ten threats to global health listed by WHO are occurring at the human-animal-environmental interfaces, comprehensive regional RA with a One Health approach made by national authorities can be a relevant added value for the global health security.

## 1. Introduction

Integrated surveillance is considered a promising working strategy [1–4] to enhance early warning of emerging infections such as arboviral diseases. In addition to providing early signals, integrated surveillance by systematically integrating multiple sources of surveillance data in a timely manner (indicator- and event-based surveillance, case-based surveillance, vector surveillance, and virus and environmental data and information) could contribute more effectively to accurate risk assessments (RA) [5]. Unfortunately, very few countries worldwide [6–8], and in the Mediterranean Region [9,10], have managed to collect and analyse surveillance data across sectors related to arbovirus transmission, and even fewer have interoperable databases. Ultimately, this limits early warning and risk assessment capacity with impact on the prevention and control of arbovirus infections. This is in line with the recognised challenges of sharing data and information, although the evidence for the public health benefits of sharing is growing with well-documented instances of an improved outcome as a result of sharing surveillance data [11–16]. Efficient data sharing also prompted an early response to the emergence of the H7N9 influenza virus in humans in China [14]; conversely, reluctance to share can hinder or slow down the response and global outbreaks have shown that inadequate surveillance and response capacity in a single country can endanger national populations and the public health security of the entire world [13]. One relevant issue is how to enhance trust within and between countries, considering that trust facilitates the sharing of data and information. Trust-building measures can take the form of face-to-face meetings, regular regional workshops, desktop exercises, joint outbreak investigations, and networking activities. These promote the sense of working towards a common goal [11].

To this aim, working with a multisectoral and transdisciplinary approach, often mentioned as a One Health approach, can help to mediate different assumptions and views and to fill knowledge gaps [17–20].

Focusing specifically on those threats which occur at the animal-human-ecosystem interfaces, several international organisations, including the World Health Organization (WHO), Food and Agriculture Organization of the United Nations (FAO), World Organisation for Animal Health (OIE), and World Bank, have recognised the critical role of multisectoral risk assessment (MRA) (multisectoral risk assessment (MRA): assessment with the concomitant participation of all the relevant sectors involved in the surveillance of a given arbovirus infection) to enhancing cross-sectoral collaboration and improving data collection and data-sharing from different sectors [21–24].

In fact, for health threats that are either emerging or existing at the interface, including food safety issues, neither the technical data nor other information important to conduct a comprehensive assessment nor the appropriate breadth of technical expertise and experience are routinely available within a single agency or sector [22].

In the dimension of capacity building and training, risk assessment exercises implemented with a multisectorial approach can foster data and information sharing across sectors reducing information gaps, highlight experiences and contributions across countries, develop the concept of a national/regional “cross-sectoral” risk assessment outcome, and guide prioritisation of actions and allocation of funds also taking into account the cross-border dimension.

In fact, regional public health threats are often presenting common characteristics such as the need of joint prevention and response activities, common coordination, and comprehensive lessons learned analysis across the actors involved, especially at borders (see the cases of Crimean–Congo haemorrhagic fever cluster in 2008 at the borders between Greece and Bulgaria [25] and in 2009 between Georgia and Turkey [26]).

These characteristics can be easily integrated in the framework of MRA.

Towards this aim, we organized three MRA exercises: one on West Nile virus (WNV) infection, one on Crimean–Congo haemorrhagic fever (CCHF), and one on Rift Valley fever (RVF) in the framework of the MediLabSecure (MLS) project [27].

The aim of these exercises was not only to formulate more reliable risk assessments but also to promote a process leading to a homogenous understanding of risk across different sectors in a given country, and across neighbouring countries, using a structured strategy of assessment. This article describes their implementation and discusses the added value of the adopted multisectoral approach.

## 2. Materials and Methods

The MLS project started in 2014 and aims at consolidating a regional network of public health institutions and laboratories, belonging to 19 non-European Union (EU) countries (Albania, Algeria, Armenia, Bosnia and Herzegovina, Egypt, Georgia, Jordan, Kosovo, Lebanon, Libya, Moldova, Montenegro, Morocco, Palestine, Former Yugoslav Republic of Macedonia, Serbia, Tunisia, Turkey, and Ukraine), for the control of zoonotic emerging viruses. It represents a cluster for awareness, risk assessment, surveillance, monitoring, and control of relevant emerging diseases, with special focus on arbovirus infections.

In this context, we designed three MRA exercises in coordination with the MLS working group and the subject-matter experts of the European Centre for Disease Prevention and Control (ECDC) and of the Italian Animal Health Institute “Istituto Zooprofilattico Sperimentale dell'Abruzzo e del Molise (IZSAM).”

For the development of the three MRA exercises, we relied on the following existing RA methodology and guidance documents: the ECDC “WNV risk assessment tool” [28], the ECDC “operational guidance on rapid risk assessment (RRA) methodology” [29], and the Food and Agriculture Organization of the United Nations (FAO) methodology of “The RVF in Niger: Risk Assessment” [30]. All mentioned tools and guidance documents were developed by subject-matter experts, had been piloted in other contexts, and were in line with the pathogens and methodological priorities identified by the MLS countries.

We invited country representatives of the human virology, animal virology, medical entomology, and public health sectors (hereby called “*sectors*”) involved in the surveillance of vector-borne diseases to participate in the three MRA exercises. Background documentation (including selected references) was sent by e-mail to participants one week before each exercise. An exercise implementation guide was also developed and sent to the facilitators together with the background documentation. The participants were asked to send national epidemiological data on the concerned pathogens that were then shared with all participants. At the start of each exercise session, participants were provided with a participant's guide.

All three exercises developed in three phases, the first always consisted in technical and methodological presentations by subject-matter experts. The second and third phases differed as shown in Table 1.

Additional details on the developed exercises, background documents, and guidance for facilitators and participants are available in the MRA exercise reports [32–34].

The added value of the multisectoral approach during the CCHF and the RVF assessments was collected by asking each participant to rank (high, medium, or low) the level of perceived benefit of the multisectoral collaboration when answering each “risk question” included in the exercises.

Pre- and posttest questionnaires, designed to assess if the MRA had increased the participant's knowledge, were prepared and submitted for the CCHF (Annex 5 of [33]) and RVF (Annex 6 of [34]) exercises. We deemed that, considering the aim of the exercises, it would have been particularly important to assess knowledge of participants on key parameters on which to rely on for the assessment, notably, surveillance data, source, and type of information and disease/infection risk factors.

Participants were also asked to compile an exercise evaluation form (Annex 6 of [34]) at the end of each exercise to provide MediLabSecure project with feedback on the quality and the pertinence of the training sessions.

### 2.1. The WNV Exercise

All the 19 countries involved in the MLS network took part in the exercise (Table 1). The participants were divided in smaller groups by country according to regional proximity.

**Figure 1:**
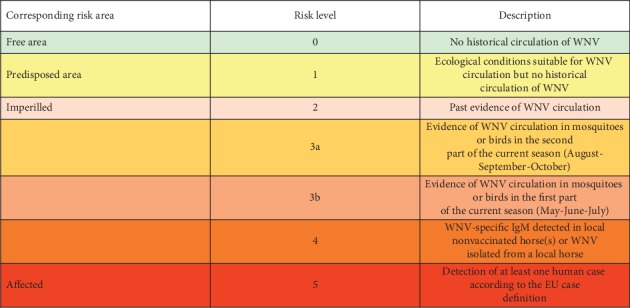
Seasonal risk levels of WNV transmission to humans with the corresponding risk area and the indicators used to define the level (source ECDC), Source: [28].

Each participant was asked to identify the risk area typology that was mostly representative of his/her country on the basis of the six risk area types defined by ECDC for WNV transmission (Figure 1).

Subsequently, the participants discussed the reasons for their identified risk area in groups, considering both national and cross-border factors. They were allowed to modify their risk area after the discussion. Then, the participants discussed in country groups the level of risk with regard to national surveillance system characteristics using the SWOT [35] analysis framework (strengths, weaknesses, opportunities, and threats analysis) guided by the ECDC tool [28]. The final risk area typology and the main aspects that had emerged from all the national SWOT analyses were presented and discussed in plenary with all other groups [31].

### 2.2. The CCHF Exercise

CCHF MRA was implemented with the countries of the Balkans and Black Sea Region of MLS (Table 1) that considered this disease as a priority for the area. The exercise was developed by adapting the information table for rapid risk assessment and the risk-ranking algorithm of the ECDC operational guidance on rapid risk assessment methodology (Annex 2 and 3 in [33]) to rate the potential of CCHF virus transmission in each participating country integrating the views of the different sectors. The assessment was done in two steps: first, the participants assessed the risk in small groups of neighbouring countries on the basis of the information delivered with the technical presentations, available national data, and the background document sent in advance; second, an assessment was made by each country over the different sectors. Each country provided the multisectoral added value to the rapporteur for plenary audience restitution.

### 2.3. The RVF Exercise

The RVF exercise was implemented with the countries of North Africa and the Middle East Region of MLS (Table 1) which considered RVF a priority for the area. The RVF exercise was developed by adapting the risk questions of the FAO RVF in Niger Risk assessment (Annex 3 in [34]) to identify the risk of RVF virus infection introduction, spread and/or persistence in each participating country. As for the CCHF exercise, the participants were divided in small groups of neighbouring countries to discuss the regional situation with the colleagues of the other sectors in the group.

For the last phase, the group was divided by country with all sectors represented because the expected outcome was the level of risk by country. Each country provided the multisectoral added value to the rapporteur for plenary audience restitution.

## 3. Results

A total of 159 participants from the 19 non-EU countries of the MLS network took part in the three exercises: 73 participants in the WNV, 42 in the CCHF, and 44 in the RVF exercise.

### 3.1. The WNV Exercise

The WNV exercise highlighted a high heterogeneity in assessing the level of risk across the involved sectors. The sharing of information and discussion between sectors and neighbouring countries reduced intersectoral variability towards a single level of risk in each country.

Each participant was provided with dots coloured as per his/her sector (i.e., yellow for human virology, blue for animal virology, green for medical entomology, and red for public health), and these dots were used to mark the identified risk area on a wall poster.

**Figure 2:**
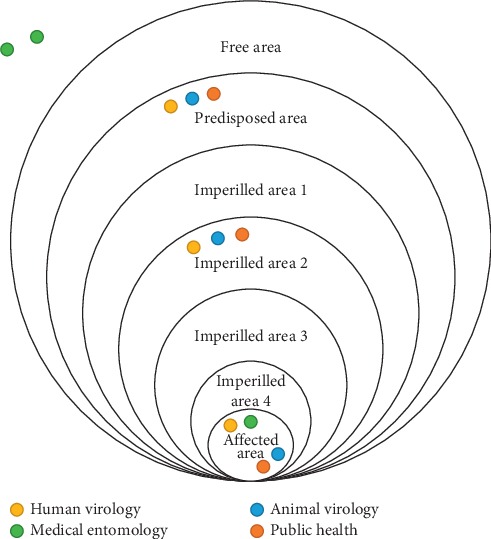
Perceived risk of West Nile virus using the ECDC risk assessment tool. Risk areas identified by three countries with consensus between sectors.

**Figure 3:**
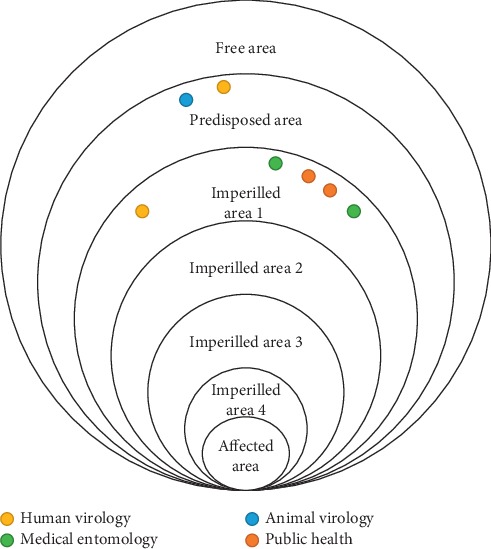
Perceived risk of West Nile virus using the ECDC risk assessment tool. Risk areas identified by two countries with less consensus between sectors.

As an example, we report here the outcomes of two groups. In Figure 2, country 1 assessed risk level 5 (affected risk area), country 2, risk level 2 (imperilled risk area), and country 3, risk level 1 (predisposed risk area) with final good agreement between different sectors within country. In Figure 3, countries 1 and 2 assessed risk level as 1 and 2, respectively, without final agreement between different sectors in one country.

The SWOT analysis underlined the critical role of integrated surveillance systems, laboratory capacity, and intersectoral collaboration for reliable risk assessments of arbovirus infections. The implementation of the first MRA exercise on WNV highlighted the need for enhancing the collaboration between sectors to reduce heterogeneity in risk assessment and for analysing the added value of a multisectoral approach.

### 3.2. The CCHF Exercise

#### 3.2.1. Knowledge and Capacity

The results of the pre- and posttests completed by thirty-five (83%) participants of CCHF exercise showed that the exercise led to improvements in the capacity to determine *risk factors* and to identify *sources of reliable information* to assess the risk. For example, with reference to the question of the test “*Would CCHF be an unusual or unexpected threat in your country?*”10 out of 35 (29%) of the respondents replied “yes” in the pretest, while in the posttest, all the respondents (35) replied “no” to this question. This suggests that the discussion between countries and the assessment exercise helped to identify possible risk factors also at cross-border or regional level (i.e., knowledge that neighbouring countries host the pathogen).

Regarding documentation for risk assessment, we reported in Table 2 the documents mentioned by the participants to assess the level of risk for CCHF in their country.

#### 3.2.2. The Added Value of the Multisectoral Approach

**Figure 4:**
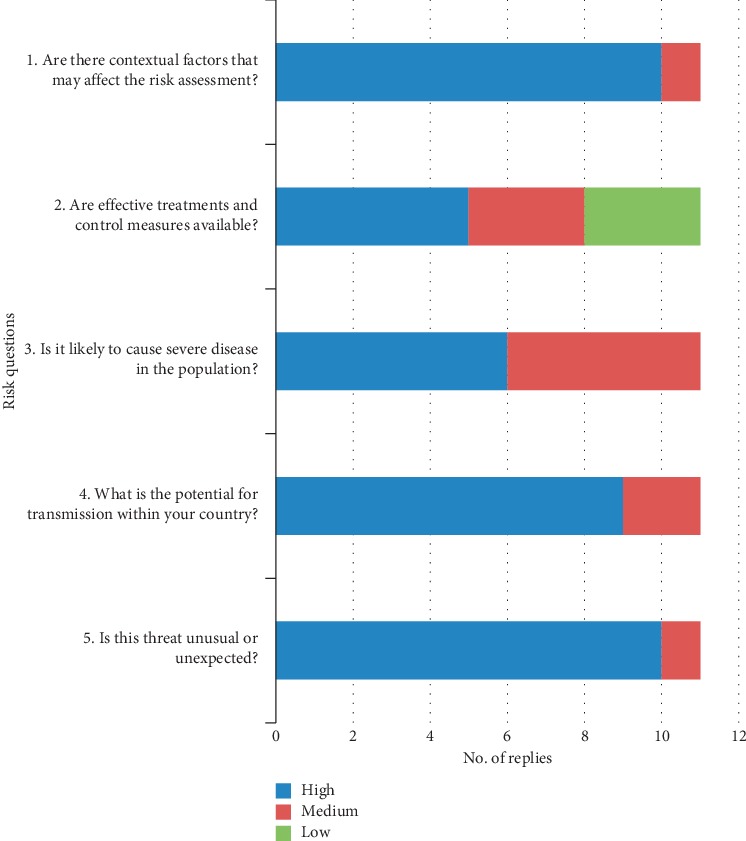
Added value of the multisectoral approach as assessed by participants to the CCHF exercise (11 countries).

The added value of the concomitant participation of several sectors to the RA for each risk question of the exercise is reported in Figure 4. These specific aspects related to the added value of the exercise were considered particularly relevant by the project's stakeholders and therefore reported in the MediLabSecure Strategic Document [31] for further developments.

The multisectoral approach was assessed as particularly valuable in “setting the scene” and in analysing comprehensively the situation having access to information and knowledge provided by each of the sectors involved in the exercise (see the added value for risk questions 1 and 5 in Figure 4 and data analysis in additional file 1).

### 3.3. The RVF Exercise

#### 3.3.1. Knowledge and Capacity

The results of the pre- and posttests, completed by twenty-one (48%) participants of the RVF exercise, showed that the exercises led to improvements in the capacity to determine risk factors. Although the participants were all able to identify several relevant risk factors, some specific risks were only identified in the posttest. Among them “*animal movements*” included by 11 (52%) and 10 (48%), as relevant risk of spread of the virus in endemic and new areas, respectively, “*social and economic instability*” included by six (29%) both as relevant risk of endemic and new areas, and “*climate changes*” included by eight (38%) and seven (33%) as relevant risk of endemic and new areas, respectively. In relation to “list kind of documents to rely on to assess the level of risk for RVF in your country,” in total, 18 (88%) and 19 (90%) of participants were able to mention kind of documents useful for RA of RVF in their countries in pre- and posttest, respectively.

### 3.4. The Added Value of the Multisectoral Approach

**Figure 5:**
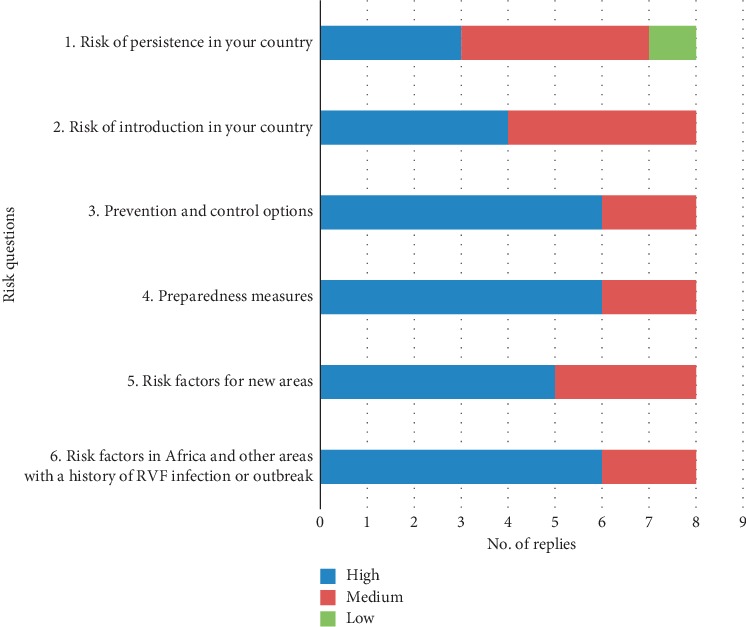
Added value of the multisectoral approach as assessed by participants to the RVF exercise (8 countries).

The country perception of the added value of the multisectoral approach is reported in Figure 5. Also, for this exercise, the multisectoral approach was particularly valuable in “setting the scene” and in analysing comprehensively the situation having access to wide range of information and knowledge provided by each of the sectors involved in the exercise (see the added value for risk questions 3, 4, and 6 in Figure 5 and data analysis in additional file 1). As for CCHF and also for RVF, the aspects related to the added value of the exercise were considered particularly relevant by the project's stakeholders and therefore reported in the MediLabSecure Strategic Document [31].

### 3.5. Results of the Evaluation of Three Exercises

Response rates to the evaluation questionnaire were 90% (66/73), 88% (37/42), and 68% (30/44) for WNV, CCHF, and RVF exercises, respectively.

Overall, 92% (WNV), 94% (CCHF), and 83% (RVF) of respondents found the exercise satisfactory. Ninety percent or more of respondents for each exercise found the discussion between sectors useful to identify the level of risk.

Almost all respondents reported that the objectives of the exercises were clearly communicated (99% for the WNV MRA exercise, 89% for the CCHF MRA, and 83% for the RFV MRS), while agreement on the appropriateness of the time allotted for the exercises was 92% for both WNV and CCHF and just 54% for the RVF exercise (see data analysis in additional file 1).

## 4. Discussion

As reported, the main aims of these exercises were to increase knowledge on MRA and raise awareness of multisectoral collaboration for conducting risk assessment of arbovirus infection with a One Health approach in the Mediterranean region. Using available tools and guidance documents allowed to avoid duplications and to refer to existing recognized published guidance.

Also, using different guidance documents helped to identify methods needed to facilitate risk assessments. For example, the WNV and CCHF exercises seem to have been facilitated by the concomitant presence of “risk questions” and algorithms in the method that guide in a stepwise manner the participants towards the final assessment. The RVF exercise instead relied only on “risk questions” to guide the participants. Replying to those questions might be difficult for people not familiar with RA methodologies and/or without access to relevant information and data. This has probably generated the perception of lack of adequate time allotted for the RVF exercise, and it is also in line with the best practice identified for a joint risk assessment by WHO-OIE-FAO [21]: “at least one member of the Joint Risk Assessment (JRA) Technical Team should have experience in risk assessment to guide the process and advise on the JRA methodology.”

Considering that different sectors may rightfully assess the risk differently, this approach has the advantage of enabling actors in each sector to recognize this variability and the reasons behind it. This awareness is a first step towards the identification of national intersectoral priorities in terms of surveillance and response that, in turn, can guide a OneHealth approach to resource allocation. In fact, MRA can facilitate prioritization of zoonosis in line with other proposed integrated approaches [19, 21, 36] and, in addition, allow joint evaluation of the risk of a specific zoonosis and prepare for a coordinated integrated response.

The pre- and posttests implemented during the exercises have highlighted that many participants did not perceive the relevance and need of recent published and unpublished documents (including those from neighbourhood countries) to support risk assessments. The exercises helped in understanding the relevance of different sources of information and data for RAs. However, it has to be noted that, in order to save time during the implementation, the ISS team searched and analysed in advance the available relevant documentation and synthetized the outcomes of the research in background documents distributed to participants. Data review was therefore not fully simulated. The identification of relevant sources of information by each of the sectors involved in the assessment and their sharing is the first step of the RA, and it should be considered among the relevant outcomes of intersectoral collaboration.

As highlighted by the WNV exercise, the multisectoral collaboration helped in the identification of the level of risk, and with the CCHF and RVF exercises, we explored at what stage of the RA this collaboration was more beneficial. Our findings suggest that the strategic added value of the multisectoral approach lies in its ability to create a common base of comprehensive and critical information, filing knowledge gaps, and to reduce uncertainty in risk assessment. This, in turn, facilitates the achievement of consensus on the comprehensive level of risk for the country taking into account the perspective of all sectors involved. The concomitant participation to the assessment of other countries of the region has also contributed to the identification of possible cross-border risk factors and to the assessment of a “regional” risk level. Similar outcomes were reported following the 2003 International Workshop [37] on the possibility, benefits, and obstacles of integration of ecological and health risk assessments based on the WHO “framework for integrated assessment of human health and ecological risks” [38]. Improved assessment quality, efficiency, and predictive capability were considered to be principal benefits of integration of risk assessments. Unfortunately, some of the obstacles to the acceptance and implementation of this approach, identified at the time, such as disciplinary and organizational barriers between disciplines, are still present. The workshop's recommendations, such as harmonization of exposure characterization, surveillance methods and models, and development of methods to facilitate comparison of risks, are still being addressed [21, 31, 39] underlying both the relevance and the complexity of the issue.

## 5. Conclusions

Increasing awareness of intersectoral priorities, including cross-border ones, through MRA is a new frontier which can support early warning capacities. This approach is relevant to reduce gaps due to unavailability of shared data and information, and it can also promote the use of multiple sources of information across sectors and facilitate consensus on operational arrangements for the RA, e.g., as recommended by the World Health Organisation (WHO) in the Western Pacific Regional Action Plan for Dengue Prevention and Control [5]. Given that six out of the ten threats to global health listed by WHO [40] are issues occurring at the human, animal, and environmental interface, the implementation of comprehensive regional assessments with a One Health approach made by national authorities using similar frameworks is promising in terms of the potential added value for the global health security agenda. This justifies further efforts in fine-tuning methodological approaches and addressing implementation challenges.

## Figures and Tables

**Table 1:** Overview of the three multisectoral risk assessment exercises conducted, Source: [31].

Exercise (place and date)	Participant countries from MediLabSecure network	Objectives	Methodology	Guidance documents
West Nile virus exercise (Paris, December 2015)	Albania, Algeria, Armenia, Bosnia and Herzegovina, Egypt, Georgia, Jordan, Kosovo, Lebanon, Libya, Moldova, Montenegro, Morocco, Palestine, former Yugoslav Republic of Macedonia (FYROM), Serbia, Tunisia, Turkey, and Ukraine	(i) Describe risk level assessment between sectors and countries (ii) Assess the cross-sectoral collaboration during the initial phase of the MediLabSecure project (iii) Make participants aware of the ECDC tool (iv) Provide indications for the next MRA exercises	(1) Map the assessment of WNV risk across four sectors (human and animal virology, medical entomology, and public health) by country and by regions (2) Conduct a SWOT analysis to assess strengths, weaknesses, opportunities, and threats in relation to the surveillance systems in place at national level, to support the risk assessment (3) Compile an evaluation questionnaire on exercise satisfaction	ECDC “West Nile virus risk assessment tool” [28]

Crimean–Congo haemorrhagic fever exercise (Belgrade, November 2016)	Albania, Armenia, Bosnia and Herzegovina, Former Yugoslav Republic of Macedonia (FYROM), Georgia, Kosovo, Moldova Montenegro, Serbia, Turkey, and Ukraine	(i) Enhance knowledge and capacity on MRA (ii) Encourage multisectoral collaboration and exchange, also among neighbouring countries and assess the related added value (iii)Provide consensus on a single national level of risk across all the sectors (iv) Make participants aware of ECDC RRA guidance and FAO RA methodology (v) Make participants aware of ECDC RRA guidance and FAO RA methodology	(1) Table0top exercise on multisector risk assessment with four sectors (human and animal virology, medical entomology, and public health) by country and by regions (2) Questionnaire on the value of multisector approach (3) Evaluation questionnaire on exercise satisfaction	ECDC “operational guidance on rapid risk assessment methodology” [29] FAO “RVF in Niger risk assessment” [30]

**Table 2:** Number of participants of the CCHF exercise who identified useful documents for RA, by type of document.

Type of document	Pretest		Posttest	
	*N* participants	Percentage	*N* participants	Percentage
No documents mentioned	9	25	2	6
Guidance, law decrees, plans	22	63	17	49
Guidance, law decrees, plans, scientific articles, unpublished documents, studies	2	6	14	39
Scientific articles	2	6	2	6
Total responders	35	100	35	100

## Data Availability

The data generated or analysed during this study, including documentation and tools prepared for the exercises, are available in the references reported in this published article (refer to [32–34]) and its supplementary information files. Pre-test and post-test forms filled in by participants are in hard copies available from the corresponding author and can be provided on reasonable request making the copies anonymous.

## Abbreviations

CCHF: Crimean–Congo haemorrhagic fever
ECDC: European Centre for Disease Prevention and Control
EU: European Union
FAO: Food and Agriculture Organization of the United Nations
IZSAM: Istituto Zooprofilattico Sperimentale dell'Abruzzo e del Molise
MLS: MediLabSecure project
MRA: Multisectoral risk assessment
RA: Risk assessment
RRA: Rapid risk assessment
RVF: Rift Valley fever
SWOT analysis: Strengths, weaknesses, opportunities, and threats analysis
WHO: World Health Organisation
WNV: West Nile virus.

## Supplementary Materials

Supplementary Materials Additional file 1: file format: excel; title of data: data analysis of the evaluation of the exercises and the added value of multisectoral approach. Description of data: replies of the participants to the questions related to the evaluation of the exercises and perceived participants' added value of the multisectoral approach.
